# The effects of different versions of a gateway STEM course on student attitudes and beliefs

**DOI:** 10.1186/s40594-018-0141-4

**Published:** 2018-10-25

**Authors:** Xiangming Wu, Jessica Deshler, Edgar Fuller

**Affiliations:** 1 Department of Mathematics and Statics, Northern Arizona University, S. San Francisco Street, Flagstaff, AZ 86011 USA; 2 Department of Mathematics, West Virginia University, P.O. Box 6310, Morgantown, WV 26506 USA; 3 Department of Mathematics, STEM Transformation Institute, Florida International University, Miami, FL 33199 USA

**Keywords:** Calculus, Persistence, Enjoyment, Confidence

## Abstract

**Background:**

Substantial research has been conducted focusing on student outcomes in mathematics courses in order to better understand the ways in which these outcomes depend on the underlying instructional methodologies found in the courses. From 2009 to 2014, the Mathematical Association of America (MAA) studied Calculus I instruction in United States (US) colleges and universities in the *Characteristics of Successful Programs of College Calculus* (CSPCC). One aspect of this study attempted to understand the impact of these courses on student experience.

**Results:**

In this paper, we describe results from an examination of the effect of course structure on students’ attitudes and beliefs across different versions of Calculus I at a large research university in the USA. To do this, we implemented a follow-up study of the national MAA study of calculus programs in part to identify potential relationships between various course structures and changes in attitudes and beliefs during the course. We compare our results both internally across these course structures and to the national data set.

**Conclusions:**

We find that the statistically significant changes measured in confidence and enjoyment exhibit differences across the different calculus implementations and that these changes are statistically independent of the underlying student academic backgrounds as shown by standardized test scores and high school GPA. This suggests that these observed changes in attitudes and beliefs relate to the experience in our varied course structures and not to the academic characteristics of students as they enter the course. In addition to our findings, we show how this national study can be used locally to study effects of courses on student affective traits.

## Introduction

From 2009 to 2014, a project led under the auspices of the Mathematical Association of America (MAA) investigated Calculus I instruction in United States (US) colleges and universities under the title *Characteristics of Successful Programs of College Calculus* (CSPCC). Results from this study showed that students’ experiences in Calculus I have significant effects on their decisions about pursuing science, technology, engineering, and/or mathematics (STEM) majors and on their beliefs and attitudes towards mathematics in general (Bressoud et al. 2013). Specifically, student experience in Calculus I has been shown to be a primary factor discouraging students from continuing in the calculus course sequence (Rasmussen and Ellis 2013). Inspired by the CSPCC study and our own offering of multiple versions of this fundamental course, we conducted a follow-up study to investigate differences and similarities in Calculus I student persistence in STEM disciplines and attitudes and beliefs towards mathematics. In this paper, we seek to address the following research question: Do different learning experiences in Calculus I influence students’ attitudes and beliefs differently? To answer this question, we measured attitudes and beliefs as in the CSPCC study and compared them across multiple course populations. We then compared the variability of student academic backgrounds across the student populations with changes in these measures to determine how changes in attitudes and beliefs were related to background data in our populations.

## Background

In 2012, the President’s Council of Advisors on Science and Technology (PCAST) reported an historically high need for STEM graduates to strengthen the national work-force (2012). Studies have shown that the rate of students pursuing a STEM degree has remained constant at about 30% (Carnevale et al. 2011; Eagan et al. 2010) nationally with less than 40% of these students actually completing a STEM degree (PCAST 2012). Calculus I, considered by many to be a gateway through which students pursuing a STEM major must pass in order to successfully pursue their degree programs, was shown by the CSPCC study to have almost a quarter of its students not receiving a passing grade (Bressoud et al. 2013). Not surprisingly, many STEM-intending students change majors (Ellis et al. 2014; Seymour and Hewitt 1997), and researchers have found a number of reasons for their departure (PCAST 2012; Seymour and Hewitt 1997) including the consistent identification of their Calculus I experience (Rasmussen and Ellis 2013; Seymour and Hewitt 1997) as a reason.

Given the Calculus I impact on student experience in STEM programs, many large-scale efforts across the USA have focused on various aspects of calculus instruction and their impact on student persistence. Researchers have considered several aspects of persistence in a number of contexts including general educational pursuits or towards the completion of coursework (Graham et al. 2013; Kuh et al. 2008; Pascarella and Terenzini 1980; Tinto 1975, 1997, 2004). In this paper, we characterize student persistence in the Calculus sequence as the primary indicator of continuing in a STEM major (Ellis et al. 2014; NCES 2014; Seymour and Hewitt 1997). According to Tinto’s (1975) framework of persistence, satisfaction in the integration of social and academic life in a community has a significant impact on persistence, and later, he asserted that this model also can be employed in the analysis of students’ learning and persistence in classrooms as communities (Tinto 1997). He highlights that this satisfaction is of critical importance to students during their freshman year because it is a time when their “membership in the communities of … campus is so tenuous” (Tinto 2004, p. 3). Most students in the USA, especially those planning to major in a STEM field, take Calculus I during their first year in college. We hypothesize that the Calculus I experiences of students in various versions of the course at our institution differ significantly and have the potential to affect their attitudes and beliefs towards mathematics as well as decisions about continuing to pursue a STEM major in different ways during this critical time.

According to the persistence frameworks developed by Graham et al. (2013) and Tinto (1975, 1997, 2004), attitudes and beliefs are critical requirements for STEM persistence. They argue that confidence and motivation are important factors associated with student persistence in a STEM major. Indeed, researchers have revealed that attitudes and beliefs play a very important role in student persistence (Graham et al. 2013; Stolle-McAllister et al. 2011; Summers and Hrabowski 2014). Specifically, many of these results show that non-cognitive factors such as motivation, interest, confidence, and beliefs are potentially important to STEM attrition (Burtner 2005; Chang et al. 2011; Espinosa 2011; Price 2010; Schoenfeld 1989; Seymour and Hewitt 1997). Students who succeed in mathematics display higher levels of enjoyment of, and persistence in, mathematics (Carlson 1999), and student achievement is significantly correlated with self-confidence and expert-like mathematical beliefs (Carlson et al. 1999). Other research has also conclusively shown that students’ beliefs and attitudes towards mathematics are strongly correlated with achievement in mathematics classes (Pajares and Miller 1995; Carlson 1999; Schommer-Aikins et al. 2005). Beliefs and attitudes have been shown to have a significant impact on problem-solving behavior (Carlson and Bloom 2005; Schoenfeld 1992), and self-efficacy and self-confidence are specifically strongly correlated with student success in the performance of problem solving (Pajares and Miller 1995).

Within these frameworks of cognition, beliefs and attitudes towards the learning process and material being learned impact the process of building understanding. As such, we must examine the role that students’ attitudes and beliefs towards mathematics, including enjoyment and confidence, play in student success in calculus. Attempting to concretely integrate these into our framework, we find that according to Leder and Forgasz (2002), there is no specific or common definition of “belief” or “attitude” since these terms “are not directly observable and have to be inferred, and because of their overlapping nature” (p.96). Other researchers hold that it is neither possible nor necessary to unify these different concepts of attitude and belief since different research problems can require different definitions (Hannula 2012; Lewis 2013). In this study, we adopt the structure formulated by Fennema and Sherman (1976) and the definition of attitude and belief, specifically that they include enjoyment and confidence as components, in their work. They formulated the definition of an attitude towards mathematics as the positive or negative emotional disposition towards mathematics and the definition of a belief towards mathematics as one’s level of psychological acceptance of the truth and value of mathematics and learning of mathematics including the usefulness, relevance, and worth of mathematics in one’s life now and in the future. With these definitions, enjoyment refers to the degree to which students enjoy working in mathematics and mathematics classes, and confidence refers to students’ confidence and self-concept of their performance in mathematics. Structurally, we note that within psychological studies (Main 2004), the notion of beliefs and values is considered to be precursors to attitude and that it is the latter that then constitutes a predisposition to action. The definitions of Fenneman and Sherman align with this structure in the sense that a student’s beliefs about mathematics will inform their attitudes by contributing to the positive or negative emotional framework for engaging in mathematical practice.

### Institutional context

The study described in this work takes place at a large research university in the USA where students can enroll in one of three different versions of Calculus I depending on their planned major and placement performance.

The *non-engineering, one-semester version* (NE) serves students primarily from science-related disciplines such as biology, chemistry, and physics. The format of the course includes highly student-focused classroom meetings with an instructor three times per week that incorporates group learning and other activities to develop strong conceptual understanding. These activities incorporate active learning approaches where students develop concepts through guided activities. Summative assessments focus on these concepts and de-emphasize complex numerical processes that would require a calculator. Students meet with graduate teaching assistants (GTAs) twice a week for additional work on problem solving and homework. During the time period for this study, there were 10 sections of this course taught with 34 students in each section. The instructors for these courses were full-time lecturers and graduate student instructors. This course used a common syllabus, common tests, and a common final exam. Instructors were allowed to modify the homework policy for their own section of the course.

The *engineering, one-semester version* (E) is built around the use of engineering-based application problems to motivate calculus concepts, and the course focuses more on technical skill development and computational precision than on deeper conceptual understanding. Three days per week, students attend a lecture meeting with the instructor. Students meet with GTAs twice a week to work on activities that often align with content they are also learning in their introductory engineering courses and that maintain a high level of computational complexity. Many of these classes are offered on the engineering school’s campus, instead of near the Department’s other classes. During the time period for this study, there were 12 sections of this course and up to 42 students in each section. The instructors for these courses were full-time lecturers and graduate student instructors. This course used a common syllabus, common tests, and a common final exam, and no modifications to policies or grading were allowed.

A third format is offered as a *two-semester Calculus I equivalent* (1A/B). Student success and placement data are used to identify a distinct cohort of students who either would have previously not been able to directly enroll in Calculus I or are at the highest risk of failing or withdrawing from a one-semester course. As a result, a primary difference for this course is the overall student population. Students learn the content covered in the standard Calculus I over a two-semester time period allowing for more in-depth coverage of core but troublesome calculus concepts and for time to review precalculus content as needed. Students meet with their instructor three times per week in a traditional lecture format with 80 students and once per week in a “laboratory” setting with a GTA and their instructor to work on activities in groups designed to support the development of concepts. The activities that students complete are a combination of paper-based and computer-supported projects. During the time period for this study, there were 4 sections of this course with 80 students in each section. The instructors for these courses were full-time lecturers. This course used a common syllabus, common tests, and a common final exam, and no modifications to policies or grading were allowed.

Each version of the course has a coordinator who supervises the instructors in that course and whose philosophy about teaching and goals for the course drive their curricular decisions independent from other courses. These courses will be referred to as versions 1A (the first half of the 1A/B sequence was the focus of our study), E, and NE for the remainder of this paper. In summary, the courses differ primarily in class size, lecture format, and recitation methods. 1A has the largest class size, followed by E, then NE. The lecture format in E and 1A is more traditional but the meeting format in NE is more active learning-driven with group discussions. Finally, the recitation activities employed in NE focus on conceptual development while those in E focus on computation and applications. The recitation activities used in 1A focus on understanding concepts using computer-supported projects. The summarized course formats for each version are shown in Table 1.

**Table 1:** Calculus I course formats

	1A	E	NE
Weekly course format	4 contact hours; 3 days of lecture with instructor; 1 day of lab with GTAs	6 contact hours; 3 days of lecture with instructor; 2 days of activities with GTAs (1.5 h each)	6 contact hours; 3 days of lecture with instructor; 2 days of activities with GTAs (1.5 h each)
Number of sections	4	12	10
Class size	80	42	34
Instructors	Full-time lecturers	Full-time lecturers, graduate students	Full-time lecturers, graduate students
Course coordination	One coordinator; common syllabus; common exams; common final exam	One coordinator; common syllabus; common exams; common final exam	One coordinator; common syllabus (minor modifications allowed); common exams; common final exam

## Methods

We collected data using two surveys administered during the CSPCC study to specifically investigate student beliefs and attitudes about mathematics among the Calculus I student population. Students received a survey between the second and third week of the fall 2015 semester and a follow-up survey 2 weeks before the end of the semester. Extra credit for completion of the surveys was given to the participating students, and each of the calculus courses had a course coordinator who determined the course’s grading scheme how they could best award extra credit for survey completion to both incentivize the process. The coordinator for E added 10 points of extra credit (worth 1% of a letter grade) to students’ final grade calculation if a student completed all surveys offered during the study. The coordinator for 1A added 2 points of extra credit to students’ final grades for completion of each survey offered during the study. The coordinator for NE added 1 point of extra credit (worth less than 0.5% of a letter grade) to students’ total quiz scores (a component of their final grade) for completing each one of the surveys.

We surveyed a total of 1019 students, and 715 students completed either the pre- or post-survey or both. We report here on the 471 respondents (120 for 1A, 246 for E, 105 for NE) who completed both the pre- and post-surveys. The response rates of the pre-survey for 1A, E, and NE are 59%, 71%, and 83%, respectively; the response rates of the post-survey for 1A, E, and NE are 42%, 42%, and 83%, respectively.

The survey questions in the instruments are mostly Likert scale prompts in multiple formats. For the 4-option Likert scale questions, the response options ranged from level “1” to level “4” and were coded with numbers from 1 to 4. For the 6-option Likert scale questions, the response options ranged from “strongly disagree” to “strongly agree” and were coded with numbers from 0 to 5. We analyzed data using factors identified and validated by the CSPCC study: beliefs, attitudes, confidence, enjoyment, and desire to continue studying mathematics (Table 2). We ran ANOVA tests to compare sample means of students’ responses on each factor in order to identify significant differences and similarities in survey responses across the three different instructional settings, instead of building a model relationship. The pre- and post-surveys provided identical statements regarding student attributes including attitudes, beliefs, mathematical confidence, enjoyment, and desire to continue to Calculus II. We compared responses to questions that appeared on both the pre- and post-surveys (Tables 5 and 6) for their total change within each course structure cohort. For each statement, we compared course population means to identify the presence of statistically significant differences in the pre- and post-survey responses.

**Table 2:** Dependent variables

Variable	Data type/source	Pre-survey	Post-survey
Beliefs	6-option Likert scale 4-option Likert scale	X	X
Attitudes	6-option Likert scale 4-option Likert scale	X	X
Confidence^a^	6-option Likert scale	X	X
Enjoyment^b^	6-option Likert scale	X	X
Desire to continue studying mathematics^c^	4-option Likert scale	X	X

^a^“I am confident in my mathematical abilities”

^b^“I enjoy doing mathematics”

^c^“If I had choice, I would never take another mathematics course/I would continue to take mathematics”

To analyze the relationship of any observed differences in the impact of the course structures, we performed a one-way repeated measures ANOVA on the levels of enjoyment, confidence, and desire for more mathematics expressed by students in the population at the pre- (time 1) and post-surveys (time 2) using the course as a three-level factor. We then attempted to distinguish the impact of student experience in these courses on response data from the influence of underlying population characteristics by performing additional one-factor ANCOVAs for the same pre- and post-survey measures with student background indicators represented by standardized measures found on the mathematics portions of the SAT or the ACT (converted to their 2015 percentiles) along with student high school GPA on the usual A = 4 to F = 0 scale drawn from institutionally reported data.

### Comparison of local data to national data

#### Student demographics

Demographic data for students in our courses for the Fall 2015 semester are shown in Table 3. One notable difference between the populations is that the NE and 1A classes have larger proportions of under-represented students. Additionally, a considerable number of students in NE take that course during their junior year compared to the E and NE courses. Our students, especially those in E, are less likely to work a full-time job compared to the national sample of research universities (Bressoud et al. 2013), and our institution has comparably fewer students from underrepresented groups.

**Table 3:** Student demographics for Fall 2015 by percentages

Student characteristic	1A *N* = 120	E *N* = 246	NE *N* = 105	National
Sex				
Female	45.83	21.55	50.48	46
Male	54.17	78.45	49.52	54
Race/ethnicity				
White	91.67	92.28	90.48	81
Black	6.67	2.85	2.86	5
Asian	3.33	2.85	8.57	17
Hispanic	1.67	1.21	0.00	9.00
College year				
Freshman	74.17	77.64	62.86	83
Sophomore	14.17	19.51	20.00	10
Junior	6.67	2.03	14.29	NA
Senior	4.17	0.00	2.85	NA
Others	0.83	0.81	0.00	NA
Enrolled full time and work > 15 h/week	8.33	5.69	8.57	9

#### Students’ academic backgrounds

In order to characterize student ability as they enter our courses, we aggregated data from their mathematics subscore on the Scholastic Aptitude Test (SAT) administered by the College Board, their mathematics subscore on the ACT exam administered by ACT, Inc. (ACT), and their high school GPA. These student academic backgrounds are shown in Table 4. We conducted an ANOVA comparison for average SAT mathematics score, ACT score (see Table 4 footnotes for descriptions), and high school GPA and found that students’ average SAT mathematics and ACT score are significantly different across three versions (*F*(2,448) = 18.823, *p* < 0.001) but that student high school GPA is not significantly different among the three versions (*F*(2,468) = 1.589, *p* = 0.205). About half of the students in each of the E and NE versions indicate that they studied calculus in high school while a lower proportion of students in 1A did. Among our students, about one fifth of the students in E took Advanced Placement (AP) Calculus (a Calculus course offered in high school in the USA intended to prepare students for an exam which can earn them college credit) in high school and subsequently passed the AP exam with a grade of 3 or higher, but very few in NE did so (AP scores of 3 or higher out of 5 can earn college credit at our institution).

**Table 4:** Students’ academic backgrounds

Student background	1A	E	NE	National
Actual institutional average SAT/ACT mathematics score				
SAT^a^	578	618	580	663
ACT^a^	25	28	26	29.1^c^
High school mathematics GPA^b^	3.62	3.65	3.64	3.77
Studied calculus in high school	35.83%	54.07%	46.67%	70%
Earned 3 or higher, AP Calculus exam	5.83%	19.51%	0.95%	26%

^a^SAT is a standardized test used for college admissions in the USA consisting of three components, and mathematics score is one of the components with a score range from 200 to 800. ACT is another standardized test used for college admissions in the USA consisting of four components, and mathematics score is one of the components with a score range from 1 to 36

^b^GPA was calculated using A = 4, B = 3, etc. for student self-reported grades

^c^This number is calculated from the original CSPCC data set

Compared to the national pool of research university students (Bressoud et al. 2013; Table 5, column 2), our students’ average SAT/ACT raw mathematics scores and high school GPA differ significantly (*p* < 0.0001). Among all three versions at our institution, the percentage of students who took calculus in high school is much lower than the national study and the percentage of students who earned a 3 or higher on the AP Calculus exam who subsequently enrolled in a college calculus class is also substantially lower. Approximately 26% of students in the national study enrolled in Calculus I had earned a 3 or higher on the AP Calculus exam. At our institution, only 11.89% of students earned a 3 or higher. However, it should be noted that students earning a score of 4 or 5 on the AP Calculus exam can earn credit for Calculus I at our institution and would therefore normally take Calculus II without taking Calculus I. Thus, students who might increase our percentage in this category would likely have earned credit for the course already and not be enrolled in Calculus I and not in our sample.

**Table 5:** Changes in sample means of students’ beliefs and attitudes

Statement		1A (mean)	E (mean)	NE (mean)	*F* ratio	*df*	*p* value
1. When studying Calculus I in a textbook or in course materials, I tend to:	Pre	2.90	3.05	3.25	3.93	2	0.02
	Post	2.81	2.95	2.77	1.55	2	0.21
2. If I had a choice, I would (/would not) continue taking more mathematics	Pre	2.61	2.98	2.52	9.77	2	0.01
	Post	2.67	2.67	2.35	3.52	2	0.03
3. My score on my mathematics exam is a measure of how well:	Pre	1.92	1.93	1.92	0.0003	2	0.10
	Post	1.97	2.47	2.48	10.78	2	0.01
4. How certain are you in what you intend to do after college?	Pre	3.14	3.09	3.20	0.67	2	0.50
	Post	3.05	3.09	3.09	0.05	2	0.95
5. The primary role of a mathematics instructor is to:	Pre	2.87	2.96	2.92	0.33	2	0.72
	Post	2.96	2.91	2.79	0.76	2	0.47
6. For me, making unsuccessful attempts when solving a mathematics problem is:	Pre	2.42	2.24	2.27	1.46	2	0.23
	Post	2.09	2.27	2.27	1.46	2	0.23
7. My success in mathematics primarily relies on my ability to:	Pre	2.53	2.64	2.64	0.55	2	0.59
	Post	2.68	2.75	2.59	1.06	2	0.35

## Results

We surveyed students’ beliefs and attitudes at the beginning (pre-survey) and end (post-survey) of the semester to collect data that might reveal differences in and changes in these beliefs and attitudes during the term as well as across the course populations.

### Changes in surveyed student attributes

As stated previously, we seek to answer the following research question: Do different learning experiences in Calculus I influence students’ attitudes and beliefs? The data revealed that a large number of students in all three versions tend to understand that trying to make sense of the materials is a better method of studying Calculus I instead of trying to memorize them (Table 5, statement 1). However, a pre- and post-survey comparison indicated a decrease in this tendency, especially in NE where there was a massive shift. In the pre-survey, we saw large differences occurred among the versions, and these differences are statistically significant according to ANOVA test. In the post-survey, the differences among three versions were smaller and not found to be statistically significant.

Students in all versions indicated a low desire to continue studying mathematics (Table 5, statement 2) unless required to do so at both the beginning and the end of semester, and we observed a decline in all versions, with a large decline in NE and an especially steep decline in E. Pairwise, the differences between 1A and E and between NE and E were large in the pre-survey but very small between 1A and NE. Results from the post-survey revealed a different picture. The differences between 1A and NE became large, but the difference between 1A and E diminished. The difference between NE and E became much smaller. ANOVA tests showed that the differences among three versions in both pre- and post-surveys were statistically significant.

Students in all versions believe exam scores measure the amount of material they understand, and there was an increase in the belief among students in E and NE that exam scores are measuring how well they can do things the way the teacher wants (Table 5, statement 3). In the pre-survey, we noticed the differences among the three versions are very small. In the post-survey, the differences across three versions become large and statistically significant according to an ANOVA test.

For statements 4 through 7, differences and similarities were observed across the three course versions in pre- and post-surveys, but the ANOVA test of the differences and similarities found no statistical significance.

Students were also asked about their confidence, enjoyment in mathematics, and desire for more mathematics (Table 6). Students across all three versions reported high levels of confidence and enjoyment of mathematics, even though they are all unexpectedly at lower levels (significant with *p* < 0.0001) than the national pool. Also, overall, students reported a statistically significant decrease in these three attributes (*p* < 0.01) from pre-survey to post-survey. This trend is consistent with the national data.

**Table 6:** Change in students’ confidence, enjoyment, and desire for more mathematics

Statement	1A (mean)	E (mean)	NE (mean)	*F* ratio	*df*	*p* value	National
Confidence							
Pre	3.57	3.84	3.80	2.67	2	0.07	4.93
Post	3.47	3.08	3.04	4.72	2	0.01	4.40
Enjoyment							
Pre	3.31	3.73	3.24	7.00	2	0.01	4.69
Post	3.26	3.13	2.74	4.25	2	0.01	4.28
Desire for more math							
Pre	2.61	2.98	2.52	9.77	2	0.01	2.97
Post	2.67	2.67	2.35	3.52	2	0.03	2.83

Differences were also observed between student populations across the three versions when specifically examining confidence, enjoyment, and desire to continue in mathematics in each version and comparing responses to these pre- and post-surveys. An ANOVA was performed on the pre-survey and post-survey results independently using course type as a factor. This approach showed that the mean of students’ confidence across three versions was statistically significantly different in the post-survey but not in pre-survey. The mean of students’ enjoyment and desire to continue in mathematics among three versions was statistically significantly different across course type in both pre- and post-surveys.

Specifically, we find that students in 1A have lower levels of confidence and enjoyment compared to NE and E. Furthermore, between E and NE, students in E show a higher level of confidence and enjoyment than those in NE. There is a small decrease in these three attributes in 1A from the pre- to the post-survey. On the other hand, we observed dramatic decreases in students’ confidence and enjoyment in E and NE. We observe a decrease in students’ desire to continue in mathematics in E, but not in NE, and the negative effects on these three attributes in E are much greater than in NE. Student beliefs and attitudes towards mathematics in 1A were observed to remain almost constant. These differences suggest that there is some impact of the course structure on these student characteristics over time, so we turn our attention to this in the next section.

### The effects of different course learning experiences

**Fig. 1:**
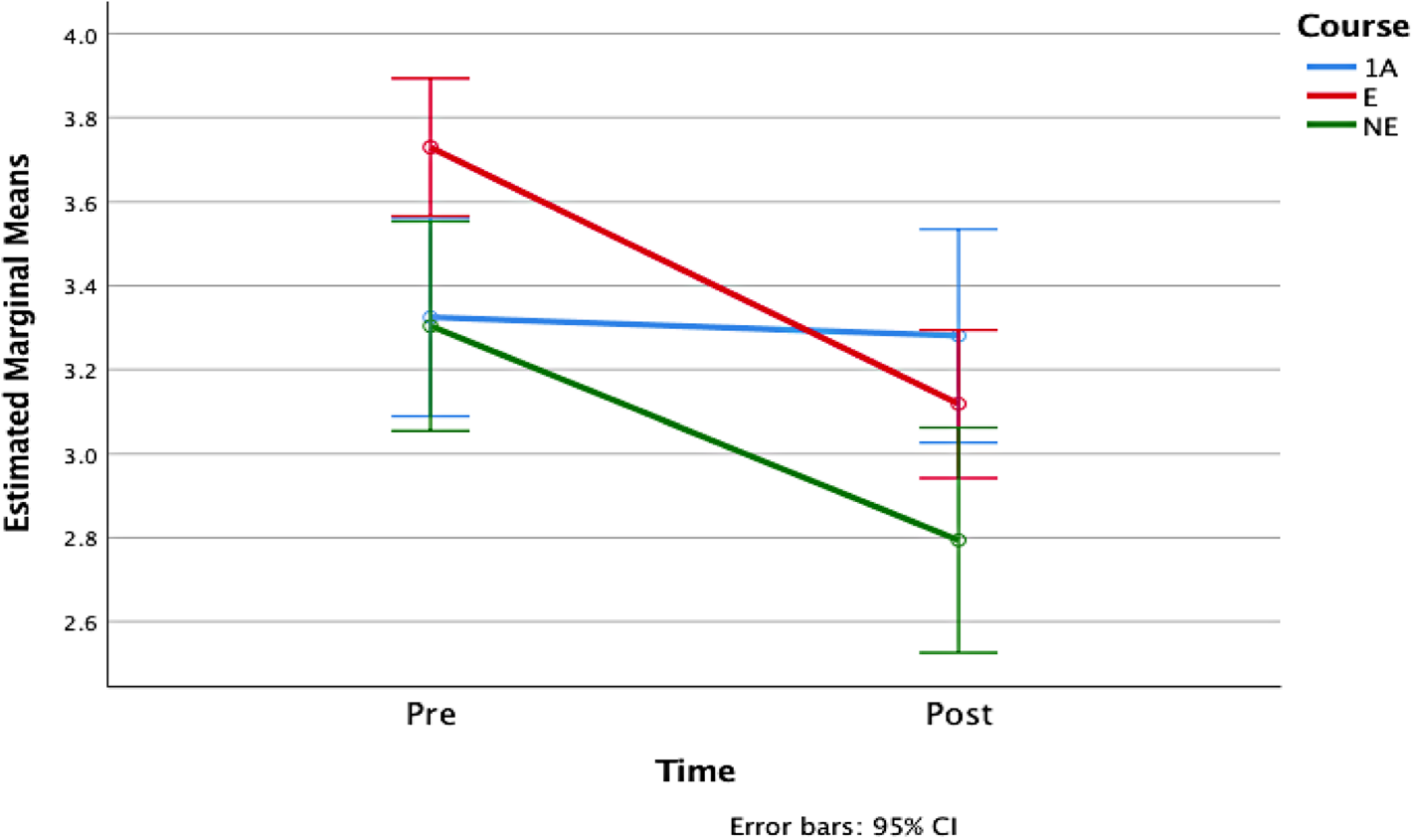
Estimated marginal means of enjoyment

**Fig. 2:**
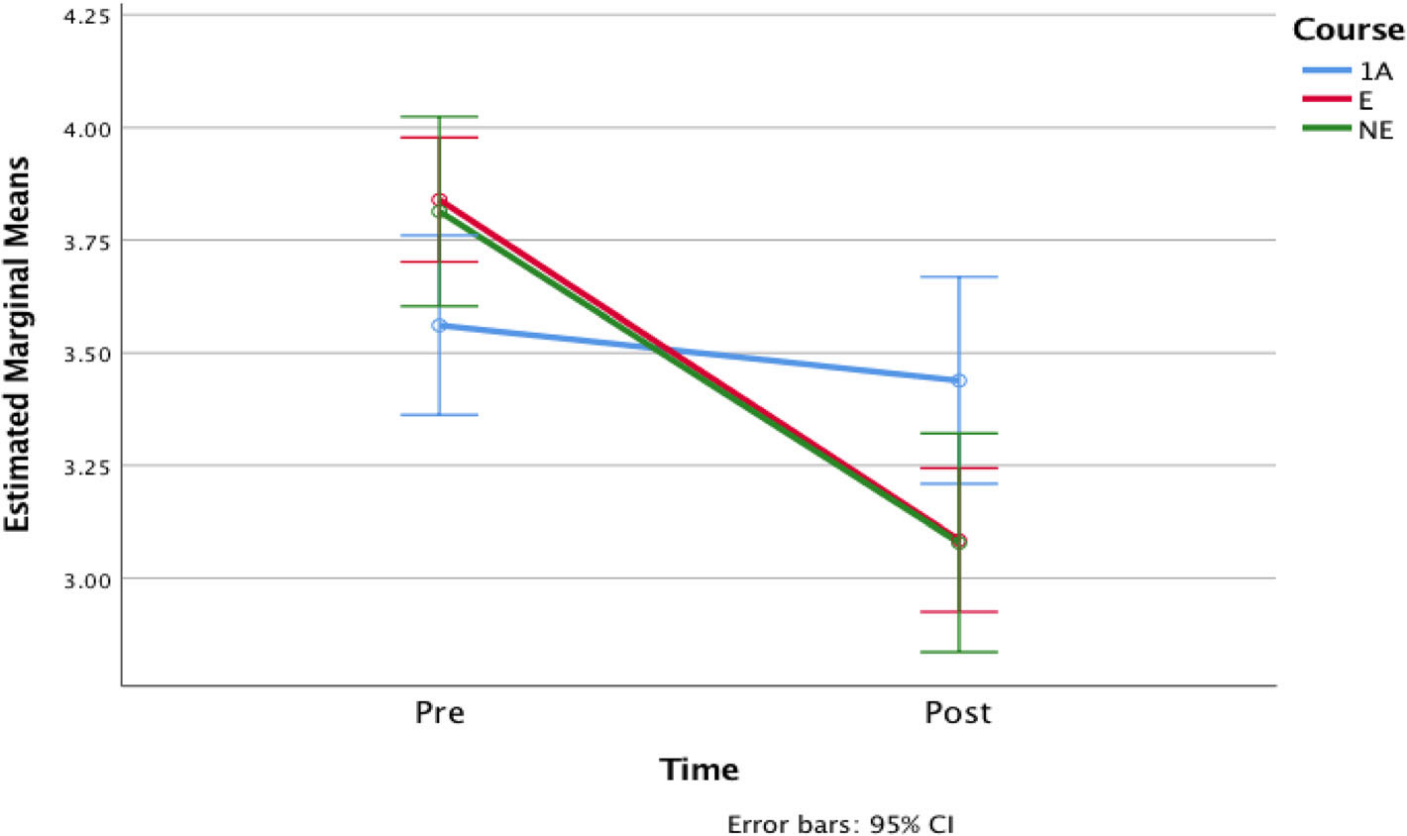
Estimated marginal means of confidence

**Fig. 3:**
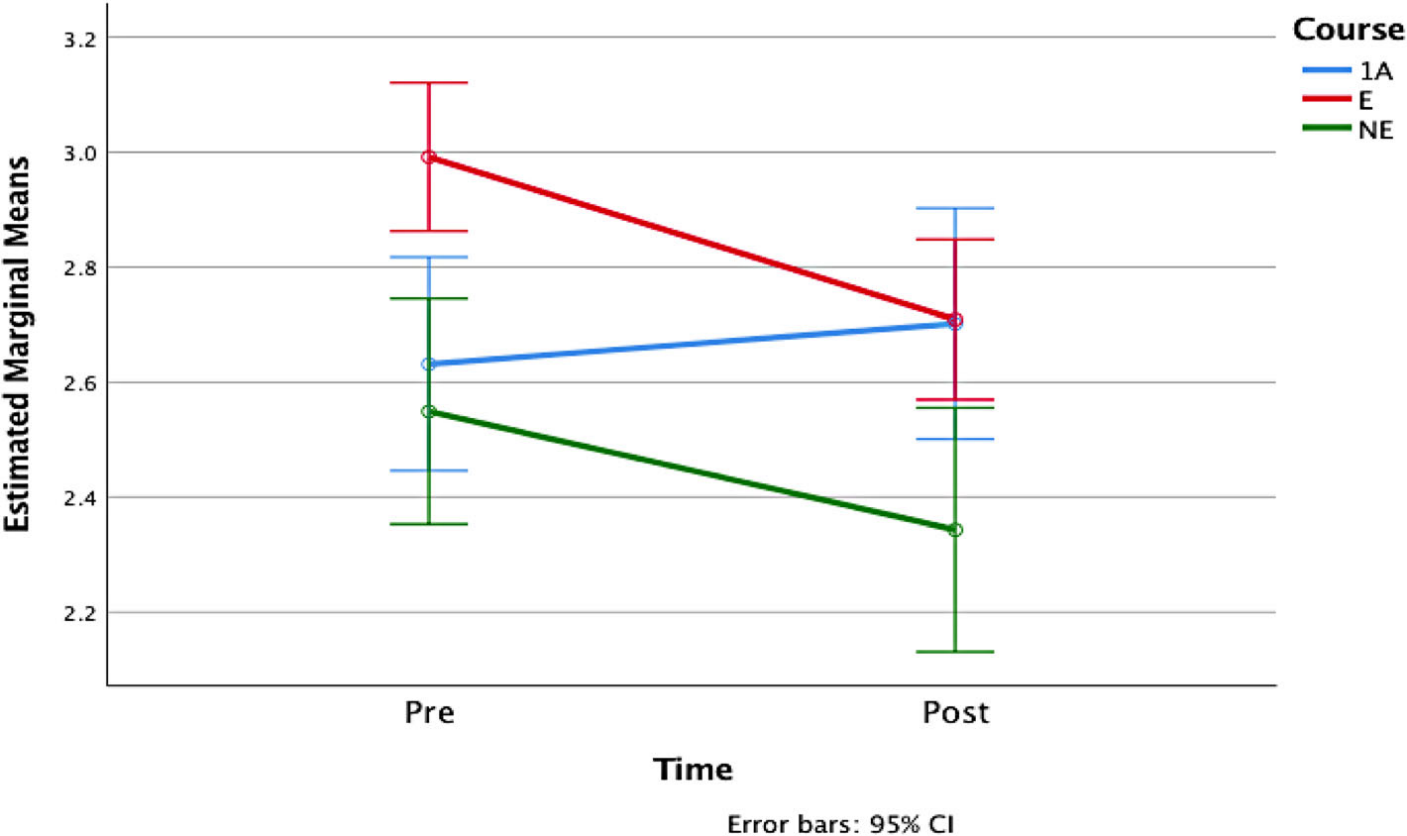
Estimated marginal means of desire

As was stated previously, one of our goals is to determine whether the observed changes in students’ attitudes and beliefs are related to the students’ learning experiences in each version of our course. The results of the prior section suggest that there are differences but care must be taken to compare the actual changes per student over time within the course groups and not just rely on differences in mean. To investigate this relationship, we applied a one-way ANOVA with repeated measures to the pre- and post-survey student response averages for questions related to enjoyment, confidence, and desire to learn more mathematics. The output from this analysis is shown in plots of the estimated marginal means of the responses with 95% confidence error bars in Figs. 1, 2, and 3. We find that the difference between course structures in the change in enjoyment is statistically significant (*F*(2, 468) = 7.304, *p* = 0.001), as is the desire to learn more mathematics (*F*(2, 460) = 4.211, *p* = 0.015) but that the difference across course structures for confidence change is not (*F*(2, 468) = 0.224, *p* = 0.8).

Next, we seek to determine if any of the variance in the measured changes in the values for enjoyment, confidence, and desire matches the variance found in the underlying demographic variables in our populations. For this, as in Bressoud et al. (2013), we convert the raw SAT and ACT mathematics subscores to the percentiles reported by the testing services for 2015 and use the percentile for each student as a covariate. We take the average of the scores if a student had scores reported for both. As was noted in an earlier section, the SAT/ACT subscores for the three populations differ significantly (*F*(2,448) = 18.823, *p* < 0.001) while GPA does not (*F*(2,468) = 1.589, *p* = 0.205), but we include GPA as an additional covariate in our tests for comparison. When populations differ, the comparisons of repeated measures can be problematic, but as observed in Schneider et al. (2015), one approach for covariates where means differ as they do here is to combine ANCOVA comparisons with ANOVA across the factor of interest (course structure here) for main effects if the covariates are re-centered by subtracting the mean in the different populations. We adjusted the SAT/ACT percentiles in this way and then performed a one-way ANCOVA with repeated measures of enjoyment and desire controlling for student ACT/SAT mathematics percentiles (recentered) as well as GPA for comparison. Course structure was still found to impact change in enjoyment and desire significantly (*F*(2, 447) = 7.288, *p* = 0.001 and *F*(2, 439) = 4.176, *p* = 0.016, respectively) when adjusted for the covariates, with between-subjects interactions insignificant for ACT/SAT and GPA in both cases.

## Discussion

As noted earlier in this work, research has consistently indicated that the affective aspects of student non-cognitive factors such as attitudes, beliefs, confidence, enjoyment, desires, and other underlying beliefs have an impact on STEM persistence (Burtner 2005; Chang et al. 2011; Espinosa 2011; Price 2010; Seymour and Hewitt 1997; Schoenfeld 1989). In the current work, we observe in the 1A format small decreases in confidence and enjoyment and a small increase in desire for more mathematics that were not significant. Students in NE also showed a decrease in desire for more mathematics, but this change was not statistically significant. The levels of these responses were not as high as was observed in the national study, and students in 1A demonstrated lower levels of agreement in confidence, enjoyment, and desire for more mathematics compared to the E and NE populations.

Our main result shows that after engaging in the E course structure, students stated a larger, statistically significant decrease in confidence, enjoyment, and desire to continue in mathematics, and in the NE course structure, they showed similarly high statistically significant decreases in confidence and enjoyment when compared to the national population. The strong difference in the response of the 1A group compared to the other two suggests that this population has different levels of enjoyment in mathematics and confidence in their mathematical abilities from the larger aggregated local calculus NE and E populations as well as from the national one-semester calculus population.

Data for E began with confidence and enjoyment levels a point lower than the national results, and the effect size we observe in the two variables, ranging from − 0.33 to − 0.70, are all larger than observed nationally. Students in NE also began with lower levels, but the effect sizes were similar to the national population. Interestingly, students in E exhibit similar levels of desire to continue as the national cohort, but the decrease in that desire is more than twice the size as that observed nationally. Students in the other two course structures enter with lower desire to continue, but their desire remains more constant; NE population’s decreases with an effect size of − 0.153 while the 1A population’s actually increased. It is reasonable to conclude that our population of students is in some way different enough from that of the national study and that an accurate determination of these differences might shed light on what aspects of our course structures resonate with our populations and which do not.

These distinctions seen from the point of view of the different course structures then suggest a similar comparison with outcomes in student behaviors. Viewed from the point of view of retention, Tinto (1975) has identified a number of areas that impact student persistence in their educational track. These can in part be characterized as facets of either academic or social integration. Examples of academic integration (Tinto 2004; Elkins et al. 2000) include grade outcomes, a student’s value of the learning process and what they learn, their enjoyment of a subject, their enjoyment or appreciation of the learning process, the level to which they identify with existing academic norms, and the level to which they identify with the role of “student.” In addition, student attitudes and beliefs impact their enjoyment and are related to their confidence in their abilities (Wesson and Derrer-Rendall 2011). In the data from this study, response rates to the two items concerning confidence and enjoyment show differences across course structures for “I am confident in my mathematical abilities” and “I enjoy doing mathematics.” Of these, the question regarding confidence shows that 7% more of the students in E respond as confident than in NE and 13% more than 1A. We expect then higher persistence of enjoyment and other beliefs for this group compared to others. Surprisingly, we see much higher negative effect sizes for E than for either NE or 1A on enjoyment, confidence, and desire for more mathematics coursework.

On the other hand, thinking of self-efficacy as our perception of our ability to deal with a situation (Pajares and Miller 1995; Ormrod 2006), attitudes and the underlying beliefs that support them tend to move towards negative or unsupportive actions when our self-efficacy is lower and so we would expect students with lower indicators of self-efficacy to exhibit larger negative changes in beliefs. That is, if we perceive ourselves as being incapable of impacting a situation such as an outcome on a mathematics exam, we tend to move away from attitudes or beliefs that support positive action such as a belief that homework/practice is valuable. With this in mind, we can look for this within our data and outcomes, and we find that, indeed, the higher negative effect size for E suggests some underlying issue with self-efficacy interacting with the course structure. Students in that course structure exhibit higher levels of self-efficacy and confidence, as expected, but these beliefs are less robust during that course than those of others again implying that indeed, the course structure itself has an impact on the students that was negative regardless of their academic background on entering it.

Our current findings focus on how student beliefs and attitudes change based on their experiences in our courses. A natural extension will be to analyze whether and how students experienced their Calculus I courses differently and to attempt to align that with the more granular differences in instruction in the three formats. In addition, it is unclear whether and how the data can be used to actually implement instructional change in the courses. The data do, however, encourage us to further investigate the reasons for explaining how these differences occurred. Looking specifically at subsets of these populations, such as only STEM-intending subgroups, sex subgroups, or STEM-persisting subgroups, may yield insights into what aspects of these courses are effective. At the very least, we hope to provide baseline data needed to document and analyze change in these factors as the courses pursue interventions to retain talented STEM majors.

Our findings represent an important first step in understanding the way in which the national results of the CSPCC study can be used to analyze the effects of a local implementation of calculus with a large population involving varied goals and backgrounds. For both the external comparison to the national data and internal comparisons within the three versions of the course offered at our institution, the small size does raise questions about the robustness of our data; if the sample size for the 1A and NE were much larger, the comparisons could be much more convincing. However, at a minimum, these findings highlight several other questions for future work: whether students do indeed benefit more long term by taking the two-semester slow-paced calculus and whether it is better to group STEM-intending and non-STEM-intending students for this coursework. This work also provides a basis for making informed decisions about changes in courses, specifically in E to address the significant decrease in confidence, enjoyment, and desire to continue in mathematics.

## Conclusion

In this report we compare students’ beliefs and attitudes towards mathematics across three different offerings of Calculus I at a single institution, and a number of differences were observed in student responses to the courses. We draw the following conclusions from this study as described below.

On the whole, prior to taking Calculus I at our institution, our students had academic backgrounds that suggested that they would be successful in our courses and reported high levels of confidence, beliefs, enjoyment, and desire for more mathematics, even though the levels of these responses were not as high as those observed in the national study. Also, students in the 1A “stretch calculus” demonstrated lower levels of agreement in these areas compared to the E and NE populations.

Focusing more on specific student beliefs and attitudes, we found dominant beliefs in the role of the instructor, the process of problem solving, and the goal of learning calculus across all three versions. These outcomes are again similar to those from the national study. In this work, however, we are more interested in any observed contrasts since our research questions focus on the differences in students’ beliefs and attitudes towards mathematics among students in the different versions of the course. As noted above, students in 1A have a lower level of confidence, beliefs, enjoyment, and desire for more mathematics, while in contrast, students in E and NE possess greater self-confidence for overcoming complications (Tables 5 and 6).

In conclusion, to answer our research question, results indicate that student experiences in three versions of Calculus I at our institution have an effect on both their beliefs and attitudes towards mathematics. Moreover, the impact of the course structures is different, and we were able to isolate the impacts within course structures from the student demographic backgrounds within those structures, implying that some aspects of the course experiences themselves are responsible for these differences. For example, we hypothesize that the strongly traditional lecture-based format of the E course led to a stronger negative impact on students’ confidence and enjoyment in mathematics though there may be other properties of the structure responsible for this effect. Further work needs to be done to determine what role the way in which engineering majors are concentrated in E and other science majors in NE might be responsible for some of these differences or the way in which student entry into the “stretch calculus” via placement impacts student experience and attitudes compared to the more mainstream courses. In all, a more precise characterization of the course formats that would allow for a quantitative comparison of the presence of active learning or the use of traditional lecturing would help the analysis of these impacts and shed further light on how such practices affect student attitudes.

## Abbreviations

1A/B: Two-semester Calculus I equivalent
ACT: ACT, Inc.
ANCOVA: Analysis of covariance
ANOVA: Analysis of variance
AP: Advanced Placement
CIP: Classification of instructional programs
CSPCC: Characteristics of Successful Programs of College Calculus
E: Engineering, one-semester version
GPA: Grade point average
MAA: Mathematical Association of America
NE: Non-engineering, one-semester version
NSF: National Science Foundation
PCAST: President’s Council of Advisors on Science and Technology
SAT: Scholastic Aptitude Test
STEM: Science, technology, engineering, and/or mathematics
